# [^64^Cu]Cu-NOTA-EV-F(ab′)_2_ Enables Same-Day Immuno-PET Imaging of Nectin-4 in Triple-Negative Breast and Urothelial Bladder Cancers

**DOI:** 10.2967/jnumed.125.270132

**Published:** 2025-09-02

**Authors:** Wenpeng Huang, Liming Li, Fangfang Chao, Qi Yang, Jason C. Mixdorf, Jonathan W. Engle, Jessica C. Hsu, Lei Kang, Weibo Cai

**Affiliations:** 1 Department of Nuclear Medicine, Peking University First Hospital, Beijing, China; 2 Departments of Radiology and Medical Physics, University of Wisconsin–Madison, Madison, Wisconsin; 3 Department of Radiology, First Affiliated Hospital of Zhengzhou University, Zhengzhou, China; 4 Department of Nuclear Medicine, First Affiliated Hospital of Zhengzhou University, Zhengzhou, China

**Keywords:** nectin-4, ^64^Cu, F(ab′)_2_ fragments, triple-negative breast cancer, bladder cancer

## Abstract

Our study aimed to develop and evaluate [^64^Cu]Cu-NOTA-EV-F(ab′)_2_ for immuno-PET imaging of nectin-4 expression in triple-negative breast cancer (TNBC) and urothelial bladder cancer (UBC) models, with the goal of achieving rapid, specific tumor targeting and high tumor-to-background contrast.

**Methods:**

Bivalent antibody fragments were generated from enfortumab vedotin (EV) using IdeS protease and conjugated with NOTA for radiolabeling with ^64^Cu. In vitro binding and uptake studies were performed using TNBC and UBC cell lines. Immuno-PET imaging and biodistribution studies were conducted in athymic nude mice bearing subcutaneous xenografts with varying nectin-4 expression.

**Results:**

[^64^Cu]Cu-NOTA-EV-F(ab′)_2_ exhibited rapid tumor accumulation and high specificity in nectin-4–positive tumors, with peak uptake observed at 4 h after injection. EV-F(ab′)_2_ demonstrated superior tumor-to-background ratios compared with the full-length EV antibody, particularly in nectin-4–expressing models (MDA-MB-468, BT474, SW780, and HT-1376). Blocking studies confirmed nectin-4–specific targeting. Favorable pharmacokinetics of EV-F(ab′)_2_ allowed for same-day imaging and reduced radiation exposure relative to intact antibodies.

**Conclusion:**

[^64^Cu]Cu-NOTA-EV-F(ab′)_2_ is a promising immuno-PET probe for assessing nectin-4 expression in TNBC and UBC.

Triple-negative breast cancer (TNBC) is an aggressive subtype that accounts for approximately 24% of newly diagnosed breast cancer cases (1). Similarly, urothelial carcinoma represents the most prevalent malignancy of the urinary tract, with urothelial bladder cancer (UBC) comprising about 90% of cases (2). Both TNBC and UBC pose significant therapeutic challenges, as current treatment strategies mainly rely on surgery, chemotherapy, and radiotherapy (3,4). Despite these interventions, many patients are diagnosed at advanced stages, underscoring the urgent need for reliable methods for early detection and longitudinal monitoring (5,6).

Nectin-4 is a calcium-independent, immunoglobulin-like adhesion molecule composed of 510 amino acids (7). Its overexpression has been strongly linked to poor clinical outcomes in patients with cancers (8–10). It is highly expressed in multiple solid tumors, including those of the lung, breast, bladder, stomach, and pancreas, where it contributes to tumor progression and increased metastatic potential (11–14). Enfortumab vedotin (EV), a Food and Drug Administration–approved antibody–drug conjugate targeting nectin-4, has shown promise in treating urothelial carcinoma. However, challenges remain in stratifying patients who are most likely to benefit from therapy and in accurately characterizing the in vivo biodistribution and pharmacokinetics of the agent (15,16).

Immuno-PET offers a noninvasive approach to visualize the in vivo expression and spatial distribution of targeted antigens (17,18). Traditional imaging probes based on full-length antibodies are often hampered by extended circulation half-lives and delayed tumor uptake, sometimes requiring days after injection to achieve optimal imaging (19). In contrast, F(ab′)_2_ fragments, lacking the Fc domain, exhibit faster blood clearance, reduced immunogenicity, and shorter half-lives, which enable lower dosages and earlier imaging windows (20).

In this study, we report for the first time, to our knowledge, the development and preclinical evaluation of [^64^Cu]Cu-NOTA-EV-F(ab′)_2_, a same-day immuno-PET imaging agent directly derived from EV. This strategy enables rapid, high-contrast visualization of nectin-4 expression, providing a clinically translatable approach for patient stratification and real-time therapeutic monitoring.

## MATERIALS AND METHODS

A comprehensive evaluation of [^64^Cu]Cu-NOTA-EV and [^64^Cu]Cu-NOTA-EV-F(ab′)_2_ was performed using various analytic methods. Nectin-4 expression in human TNBC (MDA-MB-468, BT474, and MDA-MB-231) and UBC (HT-1376, SW780, and UM-UC-3) cell lines was assessed by flow cytometry (ThermoFisher Attune) and immunofluorescence using an A1R confocal laser scanning microscope (Nikon, Inc.). Probe binding affinity and specificity were evaluated via cell uptake and binding assays.

Athymic nude Foxn1nu mice (Envigo) bearing MDA-MB-468, BT474, MDA-MB-231, HT-1376, SW780, and UM-UC-3 tumors were injected with [^64^Cu]Cu-NOTA-EV and [^64^Cu]Cu-NOTA-EV-F(ab′)_2_ (7.4–11.1 MBq/100 μL), followed by PET imaging at 1, 4, 12, 24, and 48 h after injection. Ex vivo biodistribution studies were conducted after each PET scan. Tissue sections were further analyzed by anti–nectin-4 immunostaining. Detailed experimental protocols are provided in the supplemental materials (available at http://jnm.snmjournals.org).

## RESULTS

### Preparation and Characterization of EV-F(ab′)_2_

As shown in Supplemental Figure 1A, fragmentation of the EV antibody was performed using IdeS protease, which specifically cleaves human antibodies at a single site in the lower hinge region. The Fc fragments were then separated from the F(ab′)_2_ fragments using Magne Protein A beads and MagneHis Ni particles (Promega Corporation). Subsequently, the purified F(ab′)_2_ fragments were conjugated with the NOTA chelator and labeled with ^64^Cu (Supplemental Fig. 2). The purified F(ab′)_2_ fragments have an approximate molecular weight of 100 kDa, whereas the full-length EV antibody has a molecular weight of approximately 150 kDa, as confirmed by nonreducing sodium dodecyl sulfate–polyacrylamide gel electrophoresis analysis (Supplemental Fig. 1B). High-performance liquid chromatography analysis also verified the successful preparation EV-F(ab′)_2_, as the main peak corresponding to EV-F(ab′)_2_ appears later than the EV peak (Supplemental Fig. 1C). Furthermore, immunohistochemical staining was done to evaluate nectin-4 expression in 8 normal human tissue types, in which lungs, kidneys, and intestine show obvious nectin-4 expression (Supplemental Fig. 1D).

### Assessment of Nectin-4 Expression and Cellular Binding

**FIGURE 1.**
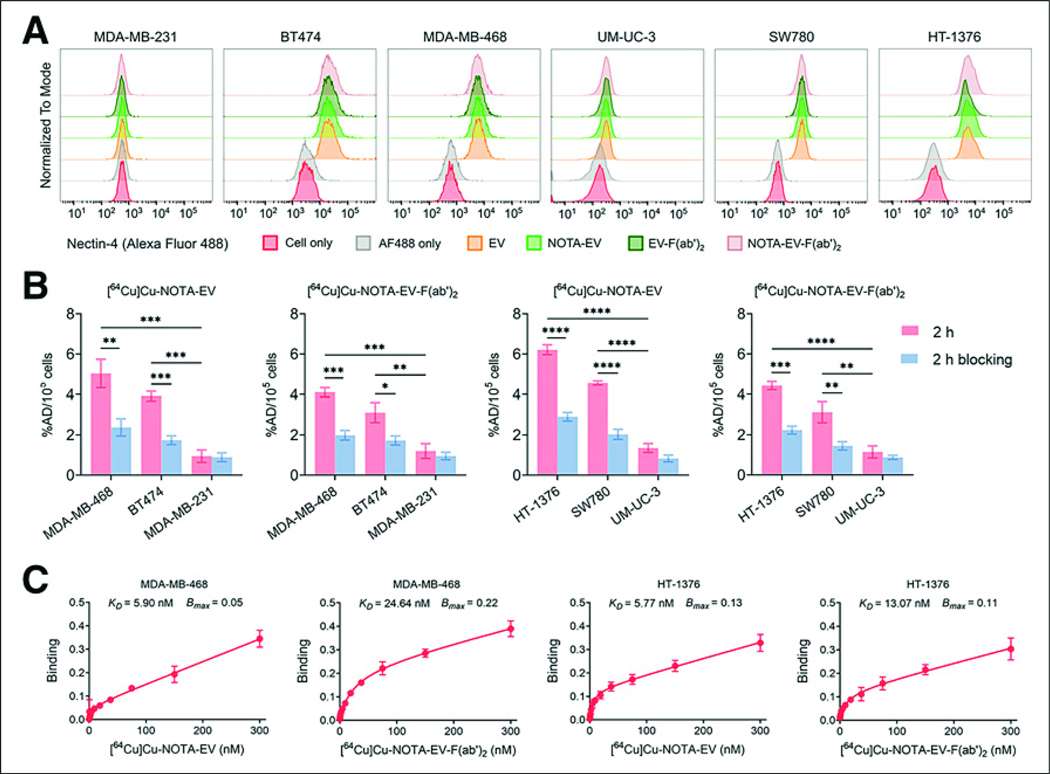
In vitro evaluation of nectin-4 expression and binding characteristics of radiolabeled EV and its antibody fragments. (A) Flow cytometry analysis of nectin-4 expression in panel of breast and bladder cancer cell lines using anti–nectin-4 antibody. (B) Cell binding of [^64^Cu]Cu-NOTA–conjugated full-length EV and F(ab′)_2_ fragments across high and low nectin-4–expressing cell lines, with and without blocking. *P < 0.05, **P < 0.01, ***P < 0.001, ****P < 0.0001. (C) Apparent binding affinity (*K*_D_) and maximum binding (*B*_max_) of [^64^Cu]Cu-NOTA-EV and [^64^Cu]Cu-NOTA-EV-F(ab′)_2_ determined by saturation binding assays in MDA-MB-468 and HT-1376 cells. %AD = percent absorbed dose; AF488 = Alexa Fluor 488.

Flow cytometry was used to evaluate the relative expression of nectin-4 in 3 human breast and 3 bladder cancer cell lines using EV as the primary antibody. Among the TNBC cell types, MDA-MB-468 has the highest expression of nectin-4 followed by BT474, whereas MDA-MB-231 has minimal expression (Fig. 1A). In terms of urothelial carcinoma, HT-1376 has the highest nectin-4 expression followed by SW780, whereas UM-UC-3 has the lowest expression. The binding affinity of EV was not affected by the conjugation of the NOTA chelator. Moreover, immunofluorescence staining results were consistent with the flow data, in which the membrane of MDA-MB-468 and HT-1376 cells have the most intense nectin-4 signal, whereas BT474 and SW780 have lower intensity of nectin-4 signal (Supplemental Fig. 3). Both MDA-MB-231 and UM-UC-3 cells have no detectable signal of nectin-4. Therefore, MDA-MB-468 and HT-1376 cells were recognized as having a high level of nectin-4, whereas BT474 and SW780 cells were identified as having an intermediate nectin-4 expression. MDA-MB-231 and UM-UC-3 cells were used as negative controls.

In vitro cellular uptake of ^64^Cu-labeled EV and EV-F(ab′)_2_ was studied in these cancer cell lines (Fig. 1B). In nectin-4–positive breast and bladder cancer cells, the uptake of [^64^Cu]Cu-NOTA-EV and [^64^Cu]Cu-NOTA-EV-F(ab′)_2_ was significantly higher than in the blocking condition. In nectin-4–negative cancer cells, no significant difference in uptake was observed between the nonblocking and blocking groups. For each tracer, the uptake in cells with high nectin-4 expression was significantly higher than in cells with intermediate or low nectin-4 expression. Furthermore, the specific binding of the full EV antibody and EV antibody fragment was determined in both MDA-MB-468 and HT-1376 cells, which have high nectin-4 expression (Fig. 1C). Binding of nectin-4 by each tracer increased with increasing concentrations and reached a plateau at approximately 50 nM. In MDA-MB-468 cells, the apparent dissociation constant values of [^64^Cu]Cu-NOTA-EV and [^64^Cu]Cu-NOTA-EV-F(ab′)_2_ were 5.90 and 24.64 nM, respectively, whereas the maximum binding capacity (B_max_) values were 0.05 and 0.22, respectively. In HT-1376 cells, the dissociation constant values of [^64^Cu]Cu-NOTA-EV and [^64^Cu]Cu-NOTA-EV-F(ab′)_2_ were 5.77 and 13.07 nM, respectively, whereas the maximum binding capacity values were 0.13 and 0.11, respectively.

### Immuno-PET Imaging and Biodistribution Studies in TNBC Models

**FIGURE 2.**
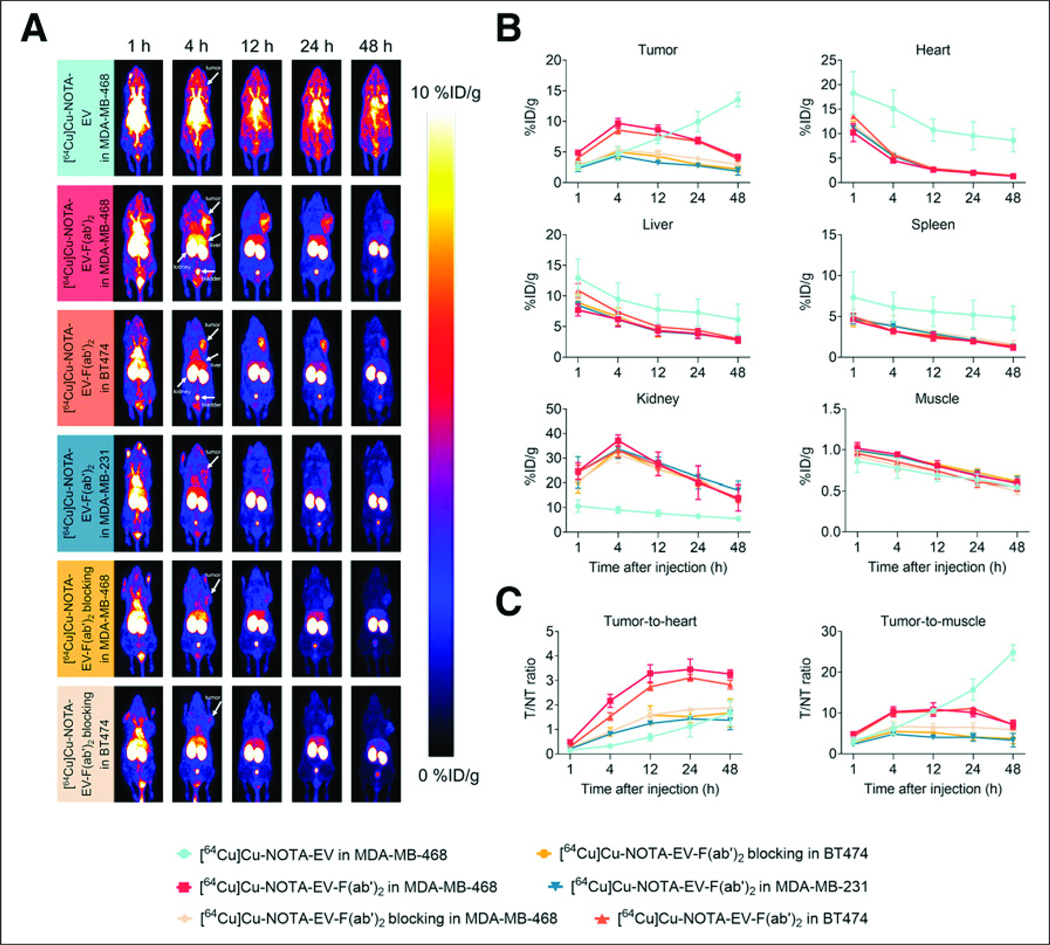
In vivo PET imaging and biodistribution of radiolabeled EV and antibody fragments in xenograft models of TNBC. (A) Representative PET images of mice bearing MDA-MB-468, BT474, and MDA-MB-231 tumors at 1, 4, 12, 24, and 48-h after injection of [^64^Cu]Cu-NOTA-EV or [^64^Cu]Cu-NOTA-EV-F(ab′)_2_, with or without blocking. Tumors are indicated by white arrows. (B) Quantitative biodistribution data (%ID/g) of radiotracers in tumor and major organs over time. (C) T/H and T/M ratios over time as determined by region-of-interest image analyses. T/NT = tumor to nontumor.

Three subcutaneous TNBC models, representing high, intermediate, and low levels of nectin-4 expression, were constructed using MDA-MB-468, BT474, and MDA-MB-231 cell lines, respectively. Radiolabeled full-length EV antibody was tested exclusively in the model with high nectin-4 expression to compare the tumor uptake and pharmacokinetic profile with those of radiolabeled EV antibody fragments. Blocking with an excess of unlabeled EV was done before the injection of radiolabeled EV antibody fragments in the murine tumor models with high and intermediate nectin-4 expression. As shown in the immuno-PET images (Fig. 2A), the uptake of [^64^Cu]Cu-NOTA-EV in the MDA-MB-468 tumor increased with time and became evident at 12 h after injection, where the uptake value was measured to be 7.20 ± 0.67 %ID/g. The highest tumor uptake value was 13.57 ± 0.95 %ID/g, observed at the final time point (48 h after injection) before sacrifice. On the other hand, the tumor uptake of [^64^Cu]Cu-NOTA-EV-F(ab′)_2_ peaked at 4 h after injection (Fig. 2B), with values of 9.70 ± 0.65, 8.57 ± 0.49, and 4.40 ± 0.45 %ID/g in breast tumors with high, intermediate, and low nectin-4 expression, respectively. The uptake in the MDA-MB-231 tumor was significantly lower than that of the MDA-MB-468 (*P =* 0.00071) and BT474 (*P =* 0.00092) tumors. When blocking was performed, the uptake values were significantly lower in the MDA-MB-468 (5.37 ± 0.48 %ID/g, *P =* 0.0016) and BT474 (5.00 ± 0.73 %ID/g, *P =* 0.0047) tumors. As expected, kidney uptake was significantly higher for the antibody fragments compared with the intact antibodies because their smaller size enabled rapid renal clearance and a much shorter circulation time.

At 12 h after injection, [^64^Cu]Cu-NOTA-EV-F(ab′)_2_ demonstrated significantly higher target-to-nontarget ratios (Fig. 2C) compared with [^64^Cu]Cu-NOTA-EV, indicating improved tumor targeting and specificity. The tumor-to-heart (T/H) ratio in MDA-MB-468 tumors reached 3.28 ± 0.29 with EV-F(ab′)_2_, markedly higher than EV (0.69 ± 0.12, *P =* 0.00031). Comparable T/H ratios were observed in BT474 (2.74 ± 0.13) group, whereas the MDA-MB-231 group showed reduced uptake (1.25 ± 0.08, *P <* 0.001) compared with MDA-MB-468 and BT474. Blocking with excess unlabeled EV significantly decreased T/H ratios in both MDA-MB-468 (1.58 ± 0.15, *P =* 0.0018) and BT474 (1.58 ± 0.31, *P =* 0.0083) tumor groups. Similarly, the tumor-to-muscle (T/M) ratio for EV-F(ab′)_2_ reached 10.87 ± 1.35 in the MDA-MB-468 group and 10.42 ± 0.64 in the BT474 group, significantly higher than 4.05 ± 0.32 in the MDA-MB-231 group (*P <* 0.01). Blocking reduced T/M ratios to 6.49 ± 0.74 (*P =* 0.0158) and 5.24 ± 0.85 (*P =* 0.0023) in MDA-MB-468 and BT474 groups, respectively. However, the intact antibody yielded a comparable T/M ratio as EV-F(ab′)_2_ in the MDA-MB-468 group (10.54 ± 0.03, *P =* 0.7447).

**FIGURE 3.**
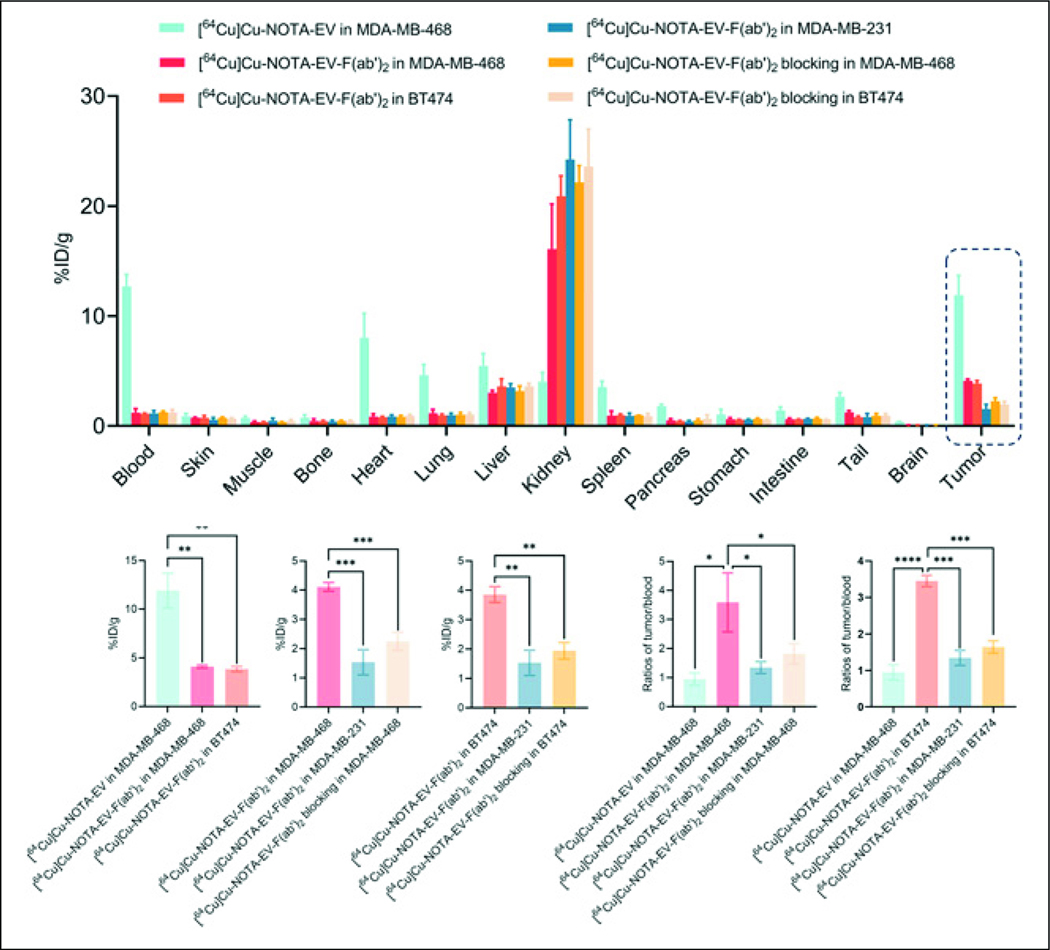
Ex vivo biodistribution and quantitative analysis of radiolabeled EV and EV-F(ab′)_2_ in TNBC models at 48 h after injection. Biodistribution of [^64^Cu]Cu-NOTA-EV and [^64^Cu]Cu-NOTA-EV-F(ab′)_2_ across major organs and tumors in mice bearing MDA-MB-468, BT474, or MDA-MB-231 xenografts. Quantitative comparisons of tumor uptake and tumor-to-blood ratios among different groups.

At 48 h after injection, ex vivo biodistribution analysis and quantitative comparisons (Fig. 3) showed the highest tumor uptake in the MDA-MB-468 group receiving [^64^Cu]Cu-NOTA-EV (11.91 ± 1.47 %ID/g). In contrast, uptake was significantly lower in the [^64^Cu]Cu-NOTA-EV-F(ab′)_2_ fragment group (4.10 ± 0.12 %ID/g, *P =* 0.0017) but was still elevated relative to the blocking control (2.25 ± 0.25 %ID/g, *P =* 0.00069). Similarly, BT474 tumors showed substantial accumulation with the EV-F(ab′)_2_ tracer (3.85 ± 0.22 %ID/g), although this was lower than that observed in the MDA-MB-468 groups receiving the intact EV tracer (*P =* 0.0015). The tumor uptake of EV-F(ab′)_2_ in the BT474 group significantly decreased on blocking (1.94 ± 0.23 %ID/g, *P =* 0.00103). Tumor uptake of EV-F(ab′)_2_ in the low nectin-4–expressing MDA-MB-231 model was minimal (1.53 ± 0.35 %ID/g, *P <* 0.005) compared with MDA-MB-468 and BT474, confirming nectin-4–specific targeting. Moreover, the T/H ratio in mice injected with [^64^Cu]Cu-NOTA-EV-F(ab′)_2_ was significantly higher than that of [^64^Cu]Cu-NOTA-EV. In MDA-MB-468 tumors, the EV-F(ab′)_2_ group reached a tumor-to-background (T/B) ratio of 3.59 ± 0.83, which was significantly greater than that of the EV group (0.95 ± 0.17, *P =* 0.0116), the MDA-MB-231 group (1.34 ± 0.17, *P =* 0.0199), and the blocking group (1.85 ± 0.32, *P =* 0.0462). A similarly elevated ratio with EV-F(ab′)_2_ was observed in BT474 tumors (3.45 ± 0.13), which also showed significant differences compared with the intact antibody in MDA-MB-468 (*P =* 0.0000793), the MDA-MB-231 group (*P =* 0.000149), and the BT474 blocking group (1.64 ± 0.14, *P =* 0.00017). These results confirm improved tumor targeting and nectin-4 specificity of the EV-F(ab′)_2_ fragments.

### Immuno-PET Imaging and Biodistribution Studies in UBC Models

**FIGURE 4.**
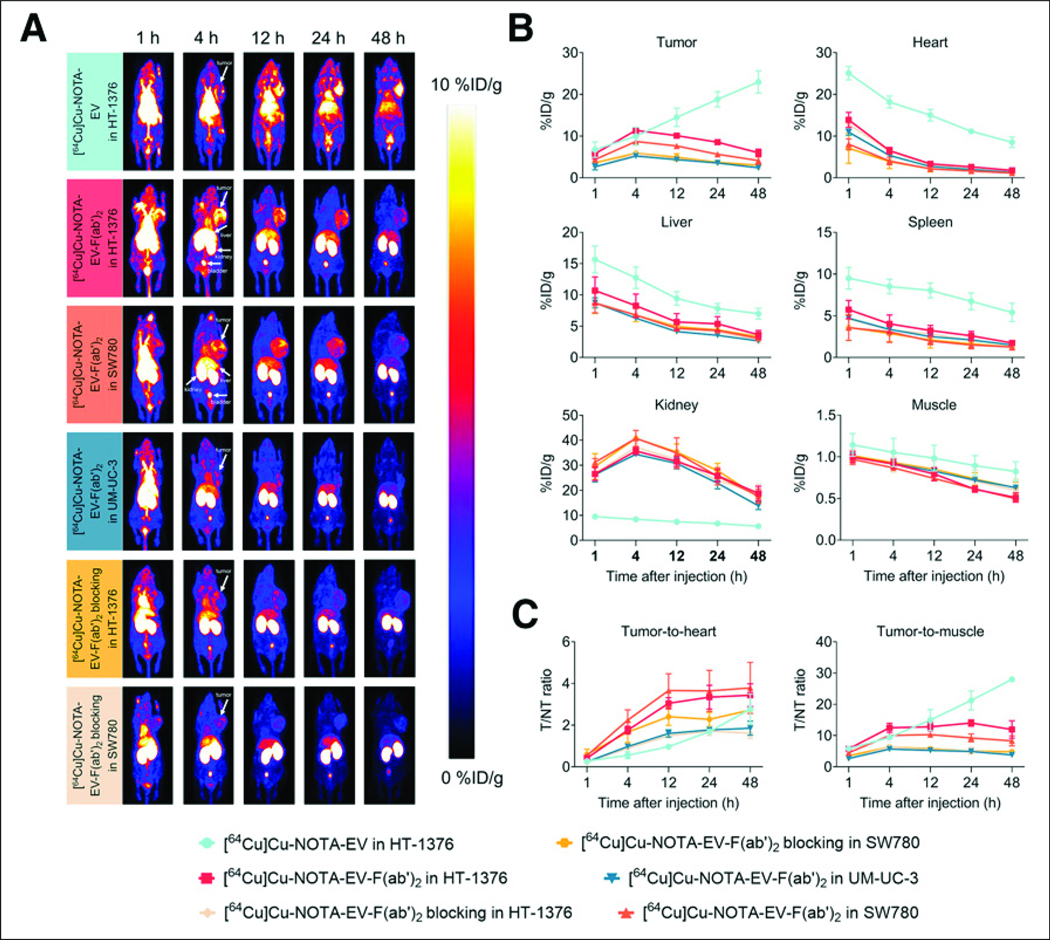
In vivo PET imaging and biodistribution of radiolabeled EV and antibody fragments in xenograft models of UBC. (A) Representative PET images of mice bearing HT-1376, SW780, and UM-UC-3 tumors at 1, 4, 12, 24, and 48 h after injection of [^64^Cu]Cu-NOTA-EV or [^64^Cu]Cu-NOTA-EV-F(ab′)_2_, with or without blocking. Tumors are indicated by white arrows. (B) Quantitative biodistribution data (%ID/g) of radiotracers in tumor and major organs over time. (C) T/H and T/M ratios as determined by region-of-interest image analyses. T/NT 5 tumor to nontumor.

Three subcutaneous UBC models, representing high, intermediate, and low levels of nectin-4 expression, were constructed using HT-1376, SW780, and UM-UC-3 cell lines, respectively. As shown in the immuno-PET images (Fig. 4A), the uptake of [^64^Cu]Cu-NOTA-EV in HT-1376 tumor increased with time and became evident at 12 h after injection, where the uptake value was measured to be 14.5 ± 1.79 %ID/g. The highest tumor uptake value was 22.97 ± 2.18 %ID/g, observed at the final time point (48 h after injection) before sacrifice. On the other hand, the tumor uptake of [^64^Cu]Cu-NOTA-EV-F(ab′)_2_ peaked at 4 h after injection (Fig. 4B), with values of 11.37 ± 0.62, 8.70 ± 0.43, and 5.20 ± 0.49 %ID/g in bladder tumors with high, intermediate, and low nectin-4 expression, respectively. The uptake in the UM-UC-3 tumor was significantly lower than that of the HT-1376 (*P =* 0.00038) and SW780 (*P =* 0.00163) tumors. When blocking was performed, the uptake values were significantly lower in the HT-1376 (5.70 ± 0.22 %ID/g, *P =* 0.00026) and SW780 (5.90 ± 0.49 %ID/g, *P =* 0.0037) tumors.

At 12 h after injection, [^64^Cu]Cu-NOTA-EV-F(ab′)_2_ demonstrated significantly higher target-to-nontarget ratios (Fig. 4C) compared with [^64^Cu]Cu-NOTA-EV, indicating improved tumor targeting and specificity. The T/H ratio in HT-1376 tumors reached 3.05 ± 0.22 with EV-F(ab′)_2_, markedly higher than EV (0.97 ± 0.10, *P =* 0.00024). Comparable T/H ratios were observed in the SW780 (3.66 ± 0.66) group, whereas the UM-UC-3 group showed reduced uptake (1.60 ± 0.14, *P <* 0.05) compared with HT-1376 and SW780. Blocking with excess unlabeled EV significantly decreased T/H ratios in both HT-1376 (1.49 ± 0.08, *P =* 0.00067) and SW780 (2.41 ± 0.34, *P =* 0.0742) tumor groups. Similarly, the T/M ratio for EV-F(ab′)_2_ reached 12.81 ± 1.11 in the HT-1376 group and 10.31 ± 0.49 in the SW780 group, significantly higher than 5.25 ± 0.63 in the UM-UC-3 group (*P <* 0.005). Blocking reduced T/M ratios to 5.44 ± 0.58 (*P =* 0.0011) and 5.78 ± 0.63 (*P =* 0.0248) in HT-1376 and SW780 groups, respectively. However, the intact antibody yielded a T/M ratio comparable to that of EV-F(ab′)_2_ in the HT-1376 group (14.98 ± 2.74, *P =* 0.3581).

**FIGURE 5.**
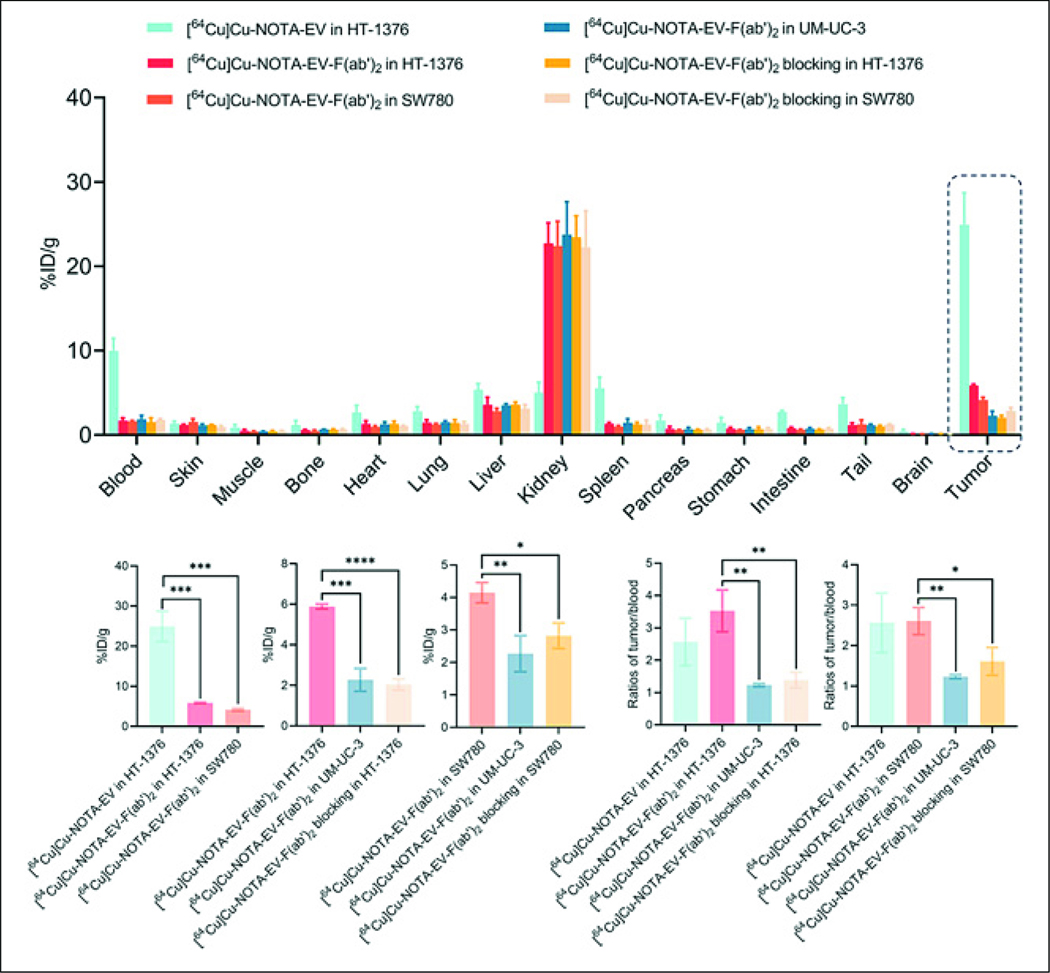
Ex vivo biodistribution and quantitative analysis of radiolabeled EV and EV-F(ab′)_2_ in UBC models at 48 h after injection. Biodistribution of [^64^Cu]Cu-NOTA-EV and [^64^Cu]Cu-NOTA-EV-F(ab′)_2_ across major organs and tumors in mice bearing HT-1376, SW780, or UM-UC-3 xenografts. Quantitative comparisons of tumor uptake and tumor-to-blood ratios among different groups.

At 48 h after injection, ex vivo biodistribution analysis and quantitative comparisons (Fig. 5) showed the highest tumor uptake in the HT-1376 group receiving [^64^Cu]Cu-NOTA-EV (24.92 ± 3.09 %ID/g). In contrast, uptake was significantly lower in the [^64^Cu]Cu-NOTA-EV-F(ab′)_2_ fragment group (5.88 ± 0.10 %ID/g, *P =* 0.00096) but was still elevated relative to the blocking control (2.04 ± 0.23 %ID/g, *P =* 0.0000255). Similarly, SW780 tumors showed substantial accumulation with the EV-F(ab′)_2_ tracer (4.15 ± 0.26 %ID/g), although this was lower than that observed in the HT-1376 groups receiving the intact EV tracer (*P =* 0.00069). The tumor uptake of EV-F(ab′)_2_ in the SW780 group significantly decreased on blocking (2.82 ± 0.32 %ID/g, *P =* 0.0104). Tumor uptake of EV-F(ab′)_2_ in the low nectin-4–expressing UM-UC-3 model was minimal (2.27 ± 0.45 %ID/g, *P <* 0.01) compared with HT-1376 and SW780, confirming nectin-4–specific targeting. Moreover, the T/B ratio was quantified for [^64^Cu]Cu-NOTA-EV-F(ab′)_2_ and [^64^Cu]Cu-NOTA-EV. In HT-1376 tumors, the EV-F(ab′)_2_ group reached a T/B ratio of 3.53 ± 0.53, which was comparable to that of the EV group (2.57 ± 0.60, *P =* 0.1638) and was significantly higher than those of the UM-UC-3 group (1.23 ± 0.04, *P =* 0.0035) and the blocking group (1.39 ± 0.20, *P =* 0.0058). The T/B ratio of EV-F(ab′)_2_ in SW780 tumors was determined to be 2.60 ± 0.27, which showed no significant differences compared with the intact antibody in the HT-1376 group (*P =* 0.9386). However, it was significantly higher compared with the UM-UC-3 group (*P =* 0.0021), and the SW780 blocking group (1.78 ± 0.40, *P =* 0.0230). These results differ from those obtained with TNBC models but confirm specificity of the EV-F(ab′)_2_ fragments toward nectin-4 expressing UBC.

### Histologic and Immunohistochemical Staining of Tumor and Normal Tissues

Hematoxylin and eosin staining of tumor xenografts derived from the aforementioned breast and bladder cancer cell lines confirmed viable tumor morphology with no signs of necrosis of hemorrhage (Supplemental Fig. 4A). Immunohistochemical staining for nectin-4 revealed strong membrane-associated expression in MDA-MB-468, BT474, HT-1376, and SW780 tumors, consistent with their known nectin-4–positive status (Supplemental Fig. 4B). In contrast, MDA-MB-231 and UM-UC-3 tumors showed minimal or undetectable staining, confirming their nectin-4–negative phenotype. To assess potential off-target expression and systemic toxicity, we further examined major organs by hematoxylin and eosin staining and immunohistochemical staining. Histologic examination showed preserved tissue architecture across various major organs with no evidence of inflammation or pathologic damage after radiotracer administration (Supplemental Fig. 4C). Corresponding immunohistochemical staining demonstrated some detectable staining in the lungs, kidneys, and intestine, suggesting low-level expression in these tissues (Supplemental Fig. 4D). All other organs showed minimal to no nectin-4 expression.

### Immunofluorescence Staining of Tumor and Normal Tissues

**FIGURE 6.**
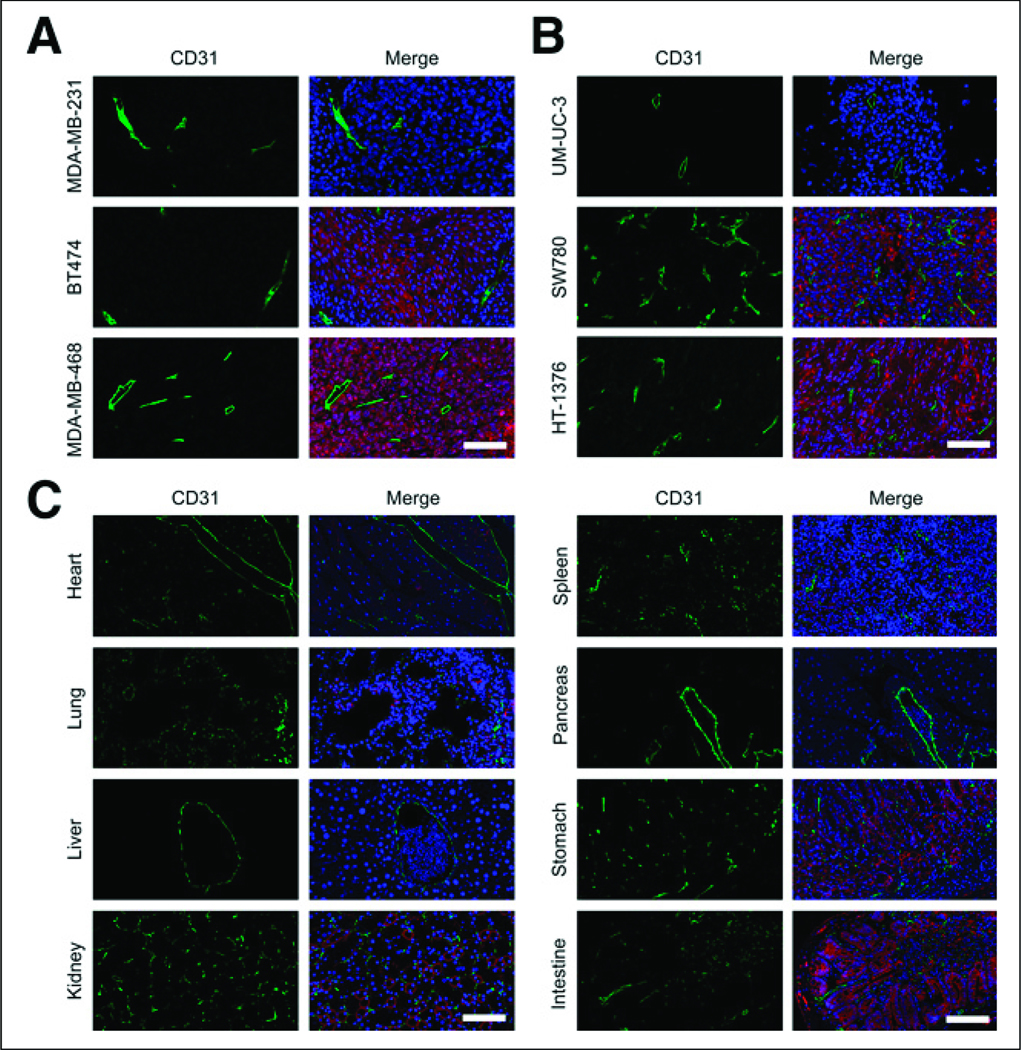
Immunofluorescence staining of nectin-4 and vasculature in tumor and normal tissues. Staining of CD31 (green), nectin-4 (red), and nuclei (blue) (A and B) in tumor sections from xenografts derived from breast and bladder cancer cell lines and (C) in various major organs.

Immunofluorescence staining was performed to assess vascularization (CD31) and nectin-4 expression in tumor and normal tissues. Tumors derived from MDA-MB-468, BT474, SW780, and HT-1376 xenografts showed abundant CD31-positive vasculature along with strong nectin-4 expression, whereas MDA-MB-231 and UM-UC-3 tumors exhibited weaker staining for both markers (Figs. 6A and 6B). In normal organs (Fig. 6C), CD31 staining revealed well-defined vascular structures across all tissues, whereas some nectin-4 expression was detected in the lungs, kidneys, and intestine, consistent with prior immunohistochemistry results.

### Extrapolation of Radiation Dosimetry to Humans

Supplemental Table 1 summarizes the estimated radiation doses to human organs from [^64^Cu]Cu-NOTA-EV-F(ab′)_2_ on the basis of biodistribution data, whereas Supplemental Table 2 provides dose estimates for [^64^Cu]Cu-NOTA-EV. For an adult woman, the calculated effective dose was 0.0353 mSv/MBq, which falls within the acceptable range for standard nuclear medicine procedures.

## DISCUSSION

Immuno-PET is a noninvasive molecular imaging technique offering high specificity, affinity, and sensitivity for antibody-based detection (21,22). Recent advances have focused on enzymatic digestion of monoclonal antibodies to produce bivalent antibody fragments, which have proven valuable in therapeutic and imaging applications (23,24). Our work seeks to evaluate antibody fragments targeting nectin-4 for immuno-PET imaging, particularly in TNBC and UBC models.

Nectin-4 is primarily expressed in the placenta, skin, lungs, and urinary system, with minimal expression in most adult tissues, making it an appealing target for cancer diagnosis and monitoring. Prior work by Campbell et al. (25) used [^89^Zr]Zr-AGS-22M6 to visualize primary and metastatic tumors, demonstrating a strong correlation between tracer uptake and nectin-4 expression. In a murine model of nectin-4–positive TNBC, uptake reached 45.3 ± 2.4 %ID/g, substantially higher than that in a murine model of nectin-4–negative TNBC (18.2 ± 2.8 %ID/g). Similarly, Ren et al. (26) labeled EV with ^124^I for noninvasive visualization of nectin-4 in UBC. [^124^I]I-EV showed high uptake in nectin-4–positive SW780 tumors (SUV_max_, 1.50 ± 0.01 at 24 h), significantly greater than in nectin-4–negative T24 tumors (SUV_max_, 0.52 ± 0.02 at 24 h; *P <* 0.001). The tracer was primarily metabolized by hepatic cells, with an optimal signal-to-noise ratio obtained at 72 h.

Despite these promising results, current immuno-PET agents suffer from slow systemic clearance and prolonged circulation times—often exceeding 3 wk—which require the use of long-lived radionuclides, such as ^89^Zr and ^124^I. These isotopes increase patient radiation exposure and delay imaging optimal imaging time points, thereby restricting clinical utility. On the other hand, small molecular tracers allow for same-day imaging. Antibody fragments such as Fab′ and F(ab′)_2_ are well suited for use with short-lived positron-emitting isotopes such as ^64^Cu and ^68^Ga, whose decay half-lives are better aligned with the rapid pharmacokinetics of these smaller constructs.

Chakravarty et al. (24) developed a ^64^Cu-labeled cetuximab Fab′ fragment via Protein A affinity chromatography, yielding improved pharmacokinetics, higher blood clearance, and greater tumor accumulation compared with unpurified fragments. Similarly, Kang et al. (23) prepared F(ab′)_2_ fragments from daratumumab using IdeS protease and verified CD38 binding through surface plasmon resonance, flow cytometry, and confocal microscopy. Immuno-PET imaging with [^64^Cu]Cu-NOTA-Dara-F(ab′)_2_ showed rapid tumor uptake at 12 h (9.5 ± 0.7 %ID/g) and a T/B ratio superior to that of the full-length Dara antibody. In our study, EV-F(ab′)_2_ and EV showed comparable, specific binding to nectin-4 as confirmed by flow cytometry and confocal microscopy. Immuno-PET with [^64^Cu]Cu-NOTA-EV-F(ab′)_2_ in TNBC and UBC models further demonstrated the feasibility of nectin-4–targeted imaging and therapy.

The choice of antibody construct has significant implications for both diagnostic imaging and therapeutic applications. From a diagnostic perspective, EV-F(ab′)_2_ fragments offer significant advantages in terms of rapid tumor accumulation and swift systemic clearance, enabling same-day high-contrast imaging while minimizing radiation exposure. When used as a therapeutic agent, the full-length EV retains its Fc region, allowing for prolonged systemic exposure and sustained tumor targeting, which is essential for effective delivery of the cytotoxic monomethyl auristatin E payload.

One limitation of our approach is the notable renal accumulation of radiolabeled antibody fragments (27), primarily resulting from lysosomal degradation, which generates radioactive metabolites that are filtered by the glomerulus and retained in renal cells (28). Although coadministration of positively charged amino acids such as ʟ-lysine has been explored to reduce renal accumulation, its effectiveness is highly variable and tracer-dependent. Alternatively, pretargeting strategies, which decouple the antibody-targeting phase from the radiolabeled probe injection, have shown promise in reducing off-target organ accumulation, including renal retention. We plan to explore these approaches in future studies to further optimize the clinical applicability of [^64^Cu]Cu-NOTA-EV-F(ab′)_2_. In this study, intact IgG was enzymatically cleaved using IdeS to produce high-purity F(ab′)_2_ fragments; however, scalable production and quality assurance under good manufacturing practices–compliant conditions remain challenging. Exploring alternative antibody formats—such as diabodies, single-chain variable fragments, Fab′ fragments, and nanobodies—which exhibit more favorable pharmacokinetics for molecular imaging, may further enhance nectin-4–targeted imaging.

## CONCLUSION

Our study demonstrated that [^64^Cu]Cu-NOTA-EV-F(ab′)_2_ Exhibits rapid, specific, and sustained accumulation in tumor tissues in TNBC and UBC models. This enables accurate, noninvasive visualization of nectin-4 expression and provides a reliable tool for monitoring tumor dynamics.

## Supplementary Material

SI

## KEY POINTS

### QUESTION:

Can F(ab′)_2_ fragments derived from EV enable rapid and specific immuno-PET imaging of nectin-4 expression in TNBC and UBC models?

### PERTINENT FINDINGS:

This study successfully developed [^64^Cu]Cu-NOTA-EV-F(ab′)_2_, which demonstrated rapid tumor accumulation, high specificity, and excellent T/B contrast in nectin-4–overexpressing TNBC and UBC models, with peak tumor uptake observed within 4 h after injection.

### IMPLICATIONS FOR PATIENT CARE:

Immuno-PET imaging with [^64^Cu]Cu-NOTA-EV-F(ab′)_2_ offers a promising approach for noninvasive, same-day visualization of nectin-4 expression, potentially improving cancer diagnosis and guiding personalized treatment strategies.
