# Supplementary material for: [64Cu]Cu-NOTA-EV-F(ab′)2 Enables Same-Day Immuno-PET Imaging of Nectin-4 in Triple-Negative Breast and Urothelial Bladder Cancers
Source: J Nucl Med. Author manuscript; Available in PMC 2026 Sep 2. (PMC12410301; doi:10.2967/jnumed.125.270132)
Supplement: Supplementary file 1 [file NIHMS2104351-supplement-SI.pdf]

Supplemental Data

Preparation of $F(ab')_2$ fragments

$F(ab')_2$ fragments of EV were prepared using the IdeS protease kit (Promega). EV is conjugated to SN-38 (a topoisomerase I inhibitor) via a cleavable linker that is attached to the Fc region of the antibody. The IdeS protease digestion process specifically cleaves the antibody below the hinge region, generating $F(ab')_2$ fragments without the Fc domain, where the drug payload is conjugated.

EV (10 mg/mL) was incubated with IdeS protease in digestion buffer (50 mM sodium phosphate, 150 mM NaCl, pH 6.6) at 37°C for 1 h with moderate shaking (650 rpm) on a ThermoShaker (Biosan, USA). Following digestion, the sample was incubated with Magne® Protein A Beads™ for 1 h on an end-over-end mixer (ThermoMixer system) to remove Fc fragments. The supernatant, containing purified $F(ab')_2$ fragments, was collected. To ensure further purification, MagneHis™ Ni Particles (Promega) was added to the digested sample, and the supernatant containing the desired $F(ab')_2$ fragments was carefully collected using a pipette. The final products were analyzed using non-reducing Sodium Dodecyl Sulfate-Polyacrylamide Gel Electrophoresis (SDS-PAGE) and high-performance liquid chromatography (HPLC) to confirm purity and integrity.

Conjugation and radiolabeling

Conjugation of S-2-(4-isothiocyanatobenzyl)-1,4,7-triazacyclononane-1,4,7-triacetic acid (p-SCN-Bn-NOTA) (Macrocyclics, Plano, USA) was carried out at pH 9.0 for 2–3 h, using a NOTA-to-EV and NOTA-to-EV-$F(ab')_2$ molar ratio of 15:1. ^{64}Cu was produced using a PET trace cyclotron (GE Healthcare, Madison, WI) via the $^{64}Ni(p,n)^{64}Cu$ nuclear reaction.

For radiolabeling, approximately 74 MBq of ^{64}Cu was diluted in 0.1 M sodium acetate buffer (pH 5.0) and incubated with NOTA-EV and NOTA-EV-F(ab')$_2$ (~200 μg) at 37°C for 1 h. The resulting [^{64}Cu]Cu-NOTA-EV and [^{64}Cu]Cu-NOTA-EV-F(ab')$_2$ conjugates were purified using a PD-10 column with PBS, and the radioactive fractions containing the [^{64}Cu]Cu-labeled conjugates were collected. Labeling efficiency and radiochemical purity were evaluated using radio thin-layer chromatography (Radio-TLC).

Cell culture

The human TNBC cell lines (MDA-MB-468, BT474 and MDA-MB-231) and bladder cancer cell lines (HT-1376, SW780 and UM-UC-3) were obtained from the American Type Culture Collection (ATCC, Manassas, VA, USA). Cell lines were cultured in Dulbecco's Modified Eagle Medium (DMEM; Gibco, USA) or RPMI 1640 medium (Invitrogen; Gibco, USA), supplemented with 10% fetal bovine serum (FBS; HyClone, USA) and 5% penicillin/streptomycin (Gibco, USA). Cells were used for in vitro or in vivo experiments when they reached 70%–90% confluence.

Animal model

All animal experiments were conducted in compliance with protocols approved by the University of Wisconsin Institutional Animal Care and Use Committee (IACUC). For tumor model establishment, four- to five-week-old female Athymic Nude-Foxn1nu mice were purchased from Envigo (Indianapolis, IN). Tumors were generated by subcutaneous injection of 4–5 × 10^6 cells, suspended in a 70 μL mixture of PBS and Matrigel (1:1, Corning, USA), into the front flank of each mouse. Tumor growth was monitored every other day, and mice were selected for in vivo experiments once tumor diameters reached 8–15 mm.

Flow cytometry

All cells were washed twice with cold PBS and adjusted to a final concentration of 1.0 × 10^5 cells/mL before analysis using the Lightning cytometer (ThermoFisher Attune). Incubation was performed using EV, EV-F(ab')$_2$, NOTA-EV, and NOTA-EV-F(ab')$_2$ at a final concentration of 50 μg/mL for 1 h on ice in the dark. Subsequently, the cells were incubated with Alexa Fluor 488 (AF488)-labeled rabbit anti-human secondary antibodies at room temperature (RT) for 0.5 h. Data acquisition and analysis of mean fluorescence intensities were performed using FlowJo software.

Cell uptake experiments

Cells were seeded in 24-well plates at a density of 1.0 × 10^5 cells per well, followed by the addition of 1 mL of serum-free medium containing 37 kBq of [^{64}Cu]Cu-NOTA-EV and [^{64}Cu]Cu-NOTA-EV-F(ab')$_2$. The plates were incubated at 37°C for 2 h time points. After incubation, 1 mL of 1 M NaOH was added to each well for cell lysis, and the lysates were collected. Non-radiolabeled EV-F(ab')$_2$ fragments were used as the specific blocking agent to competitively inhibit the binding of [^{64}Cu]Cu-NOTA-EV-F(ab')$_2$ to Nectin-4 receptors. The final concentration of non-radiolabeled EV-F(ab')$_2$ used was 10 μg/mL, ensuring effective receptor saturation during the incubation period. The radioactivity in both the supernatants and lysates was measured using an automatic gamma counter (Wizard2, PerkinElmer).

Cell binding assay

For binding studies, MDA-MB-468 cells and HT-1376 cells were plated in a 96-well filter plate at a density of 1.0 × 10^5 cells per well. A range of [^{64}Cu]Cu-NOTA-EV and [^{64}Cu]Cu-NOTA-EV-F(ab')$_2$ solutions with final concentrations between 0.15 and 300 nM were prepared, and cells were incubated with these solutions at 37°C for 4 h. After incubation, the cells were thoroughly washed, and radioactivity was measured using a gamma

counter. Surface Nectin-4 expression, apparent dissociation constant (K_D), and maximum binding capacity (B_{max}) were determined using total and nonspecific binding data analyzed with GraphPad Prism software, with the K_D value largely depends on the surface density of Nectin-4 expression on the cell system which is used for affinity measurements.

Immunofluorescent cell staining

For the immunofluorescent staining assay, cells were plated in 35 mm confocal culture dishes at a density of 2.0 × 10^5 cells and incubated overnight. Following fixation with 3% paraformaldehyde at 4°C, the cells were treated with EV (1:800), washed three times with cold PBS, and subsequently incubated with Alexa Fluor 488-labeled rabbit anti-mouse secondary antibodies at RT for 30 minutes. After three additional washes with cold PBS, the cells were stained using a DAPI-containing mounting medium. Coverslips were then placed, and imaging was conducted using a Nikon A1R confocal laser scanning microscope (Nikon, Inc., Melville, NY).

ImmunoPET imaging

PET imaging studies were conducted using an Inveon microPET scanner designed for rodents (Siemens Medical Solutions USA, Inc.). Tumor-bearing mice received an intravenous injection of 7.4–11.1 MBq of [^{64}Cu]Cu-NOTA-EV and [^{64}Cu]Cu-NOTA-EV-F(ab')$_2$, and imaging was performed at 1, 4, 12, 24, and 48 h post-injection (p.i.). Images were calibrated to display signals within a range of 0–10% of the injected dose per volume, expressed as %ID/g. For blocking studies, mice were injected with an excess of unlabeled EV-F(ab')$_2$ (2 mg) 0.5 h before the experiment. Quantitative analysis involved delineating regions of interest (ROI) in the heart, liver, kidneys, muscles, spleen, and tumors. Tumor-to-heart (T/H) and tumor-to-muscle (T/M) ratios were subsequently calculated to assess tracer distribution.

Ex vivo biodistribution

After the final imaging session, mice were euthanized via carbon dioxide asphyxiation. Key organs and tissues, including the tumor, heart, liver, spleen, lungs, kidneys, stomach, intestines, tail, muscles, bones, brain, skin, and blood, were excised and weighed. The radioactivity of each sample was quantified using an automated gamma counter, and biodistribution was expressed as %ID/g.

Radiation dosimetry prediction

Balb/c mice (female, 4–5 weeks old) were divided into 4 groups (n = 3). Balb/c mice were then injected with 0.74 MBq of [^{64}Cu]Cu-NOTA-EV and [^{64}Cu]Cu-NOTA-EV-F(ab')$_2$ (100 μL) through the tail vein and sacrificed 1, 4, 24 and 48 h after injection. The extrapolated doses to adult females were calculated using decay corrected ^{64}Cu biodistribution data. The dosimetry analysis was performed using the OLINDA/EXM dose-spherical model provided by the OLINDA/EXM software.

Histological analysis

Histological assessments included hematoxylin-eosin (H&E) staining, immunohistochemistry (IHC), and immunofluorescence. Immunofluorescent staining was conducted on tumor tissues and major organs, including the heart, lungs, liver, spleen, stomach, pancreas, intestine, and kidneys, to evaluate Nectin-4 expression according to established protocols. Primary antibodies used were anti-human Nectin-4 (1:300, Abcam) and mouse anti-human CD31 (1:300, Servicebio). Secondary antibodies consisted of AF488-labeled goat anti-rabbit IgG (1:200, Servicebio) and Cy3-labeled goat anti-mouse IgG (1:200, Servicebio). Tissue morphology and staining patterns were analyzed and captured using a NIKON Eclipse Ti confocal microscope.

Statistical analysis

Quantitative data are expressed as mean ± standard deviation (SD). Statistical analyses were conducted using the Student's t-test or one- or two-way ANOVA in GraphPad Prism version 8.0. A P-value < 0.05 was considered statistically significant. All experiments were performed in triplicate or more, with results presented as mean ± SD unless specified otherwise. Statistical significance in the figures is denoted by asterisks: * P < 0.05; ** P < 0.01; *** P < 0.001; **** P < 0.0001.

Supplementary results

Preparation of $F(ab')_2$ fragments

As shown in **Supplemental Figure 1**, the fragmentation of EV antibody was performed using IdeS protease, which specifically cleaves human antibodies at a single site in the lower hinge region. The Fc fragments were then separated from the $F(ab')_2$ fragments using Mange® Protein A beads and MagneHis™ Ni Particles. Subsequently, the purified $F(ab')_2$ fragments were conjugated with the NOTA chelator and labeled with copper-64 **(Supplemental Figure 2)**. Furthermore, immunohistochemical staining was done to evaluate Nectin-4 expression in eight normal human tissue types, where lungs, kidney, and intestine show obvious Nectin-4 expression.

In EV, the MMAE cytotoxic payload is primarily conjugated to cysteine residues within the Fc region via cleavable maleimide linkers. The IdeS protease cleavage selectively removes the Fc portion, thereby eliminating most of the attached MMAE from the resulting $F(ab')_2$ fragments. This process significantly diminishes concerns regarding residual cytotoxicity in the imaging probe. Future studies will include direct mass spectrometric analysis to further confirm the absence of MMAE in the final product.

SDS-PAGE analysis showed that while EV appeared as a single band around 150 kDa, the EV-$F(ab')_2$ sample presented as a cluster of closely migrating bands around 100–130 kDa, which we attribute to mixed bands arising from microheterogeneity. A minor shoulder

preceding the main peak was observed, likely attributable to a small amount of residual undigested full-length EV or the presence of non-covalent F(ab')2 aggregates. While the current purification strategy yields sufficient purity for preclinical imaging studies, additional refinement could be applied in future studies to achieve clinical-grade purity if required.

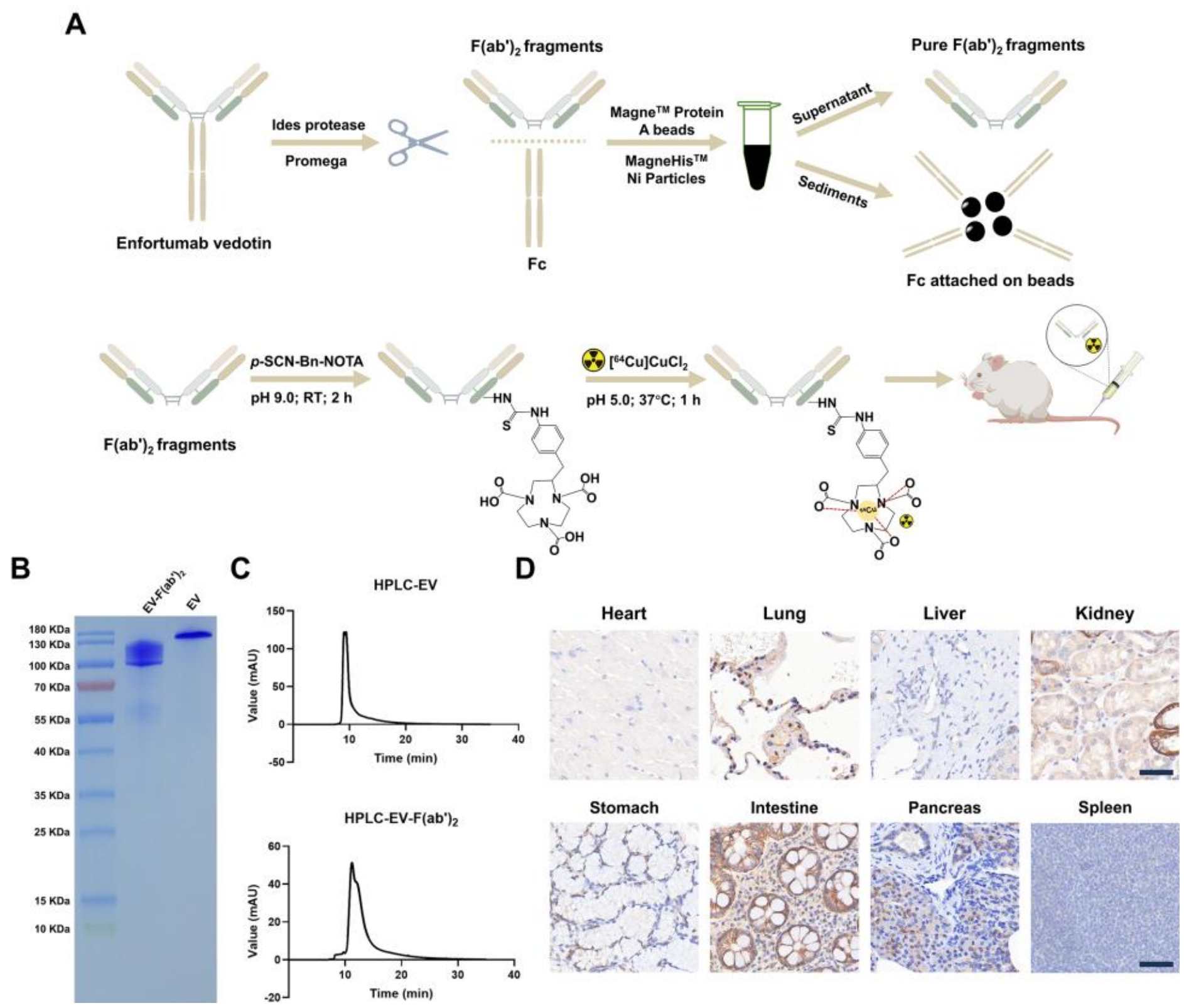

Supplemental Figure 1. Preparation of antibody fragments derived from EV. (A) Schematic illustration of the antibody fragmentation and purification workflow, followed by NOTA chelator conjugation and radiolabeling of EV-F(ab')2 with the Cu-64 radioisotope. **(B)** Non-reducing SDS-PAGE and **(C)** HPLC analyses confirm the successful generation and purity of EV antibody fragments. **(D)** Immunohistochemical staining reveals Nectin-4 expression across various organs.

Radiosynthesis of [^{64}Cu]Cu-NOTA-EV-F(ab')$_2$

Radiolabeling with $^{64}CuCl_2$ at pH 5.0 and 37°C for 1 h resulted in a high radiochemical yield (85.40 ± 2.43, n = 5), as verified by radio-HPLC (**Supplemental Figure 2A**). Following purification with PD-10 columns, the radiochemical purity (RCP) of the tracers surpassed 99%, with no detectable free ^{64}Cu in the target fractions of [^{64}Cu]Cu-NOTA-EV-F(ab')$_2$ **(Supplemental Figure 2B**). In vitro stability studies confirmed that [^{64}Cu]Cu-NOTA-EV-F(ab')$_2$ maintained a high RCP (> 98%) after 24 h of incubation in 0.01 M PBS and 5% human serum albumin (HSA), demonstrating excellent stability and suitability for further research applications **(Supplemental Figure 2C and 2D**). Additionally, the measured specific activity of [64Cu]Cu-NOTA-EV-F(ab')2 was approximately 0.36 ± 0.02 MBq/μg (n = 5).

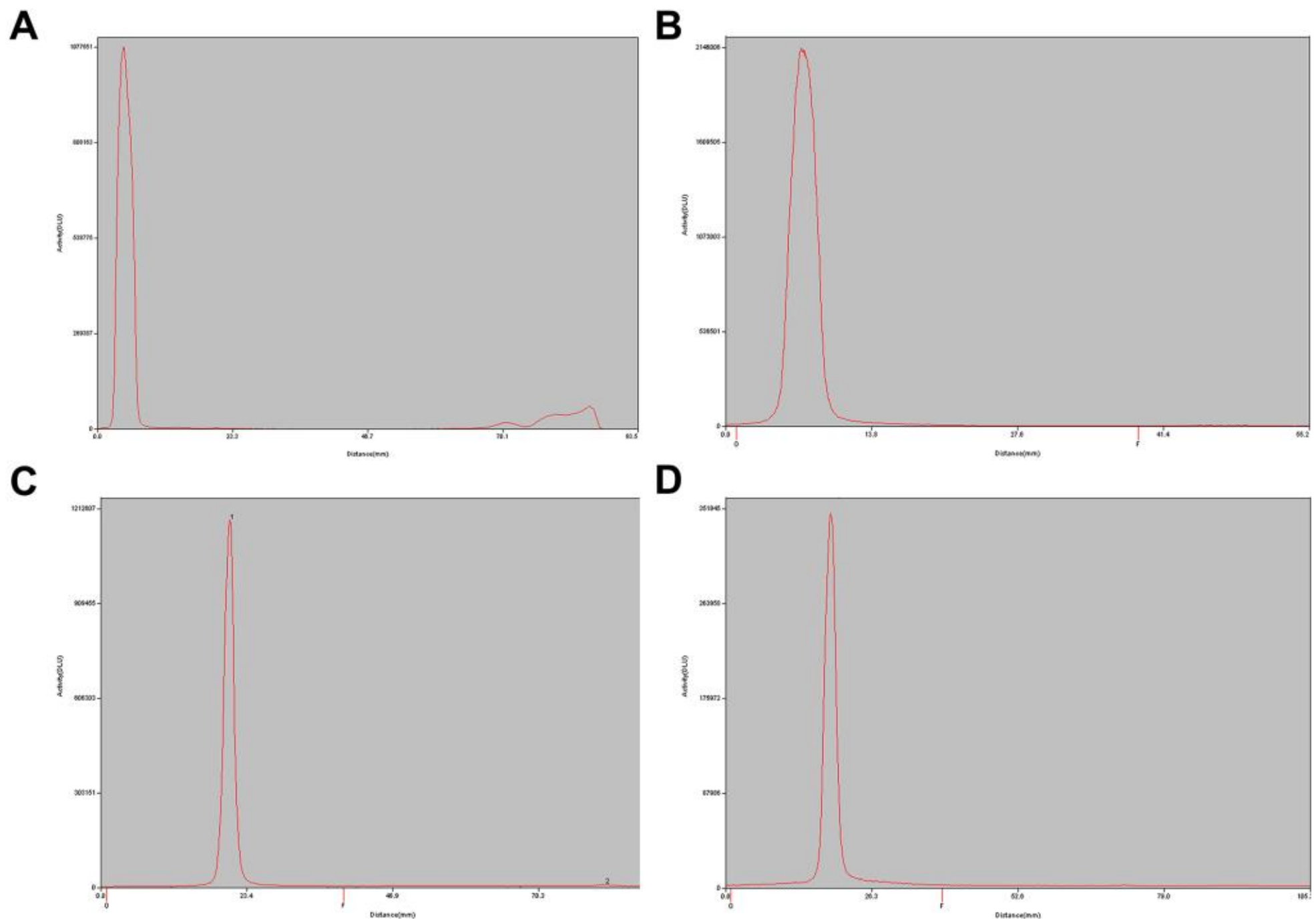

Supplemental Figure 2. Radio-TLC analysis of the radioactive assay utilizing [^{64}Cu]Cu-NOTA-EV-F(ab')$_2$ as the substrate. (A) Radiochemical yield of [^{64}Cu]Cu-NOTA-EV-F(ab')$_2$. **(B)** Radiochemical purity of [^{64}Cu]Cu-NOTA-EV-F(ab')$_2$ following purification with a PD-10 column. **(C-D)** Radiochemical purity of [^{64}Cu]Cu-NOTA-EV-F(ab')$_2$ after 24 h of incubation in 0.01 M PBS and 5% human serum albumin.

Immunofluorescence staining

Immunofluorescence staining results were consistent with the flow data, where the membrane of MDA-MB-468 and HT-1376 cells have the most intense Nectin-4 signal, while BT474 and SW780 have lower intensity of Nectin-4 signal **(Supplemental Figure 3)**.

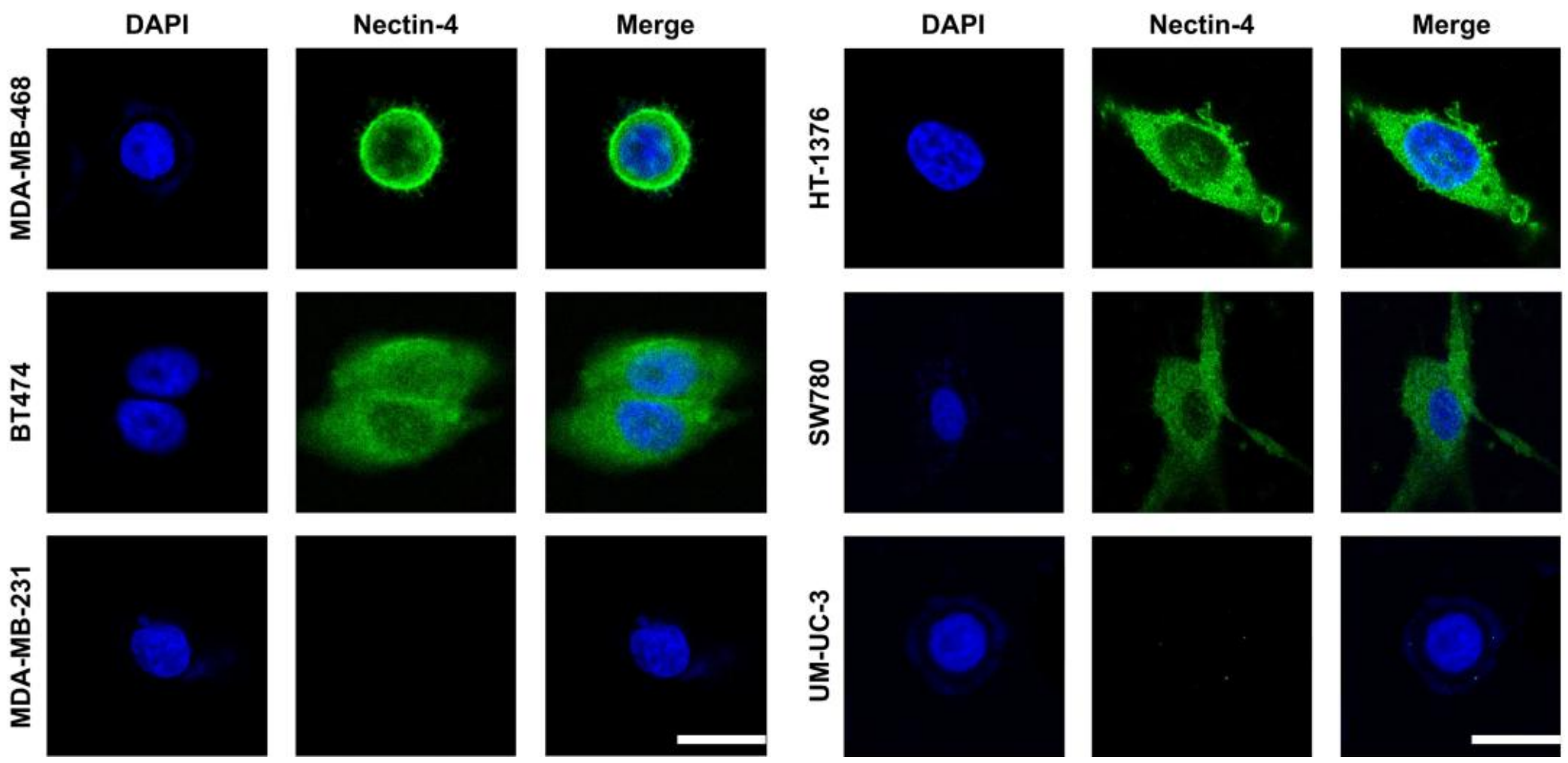

Supplemental Figure 3. Immunofluorescence staining of Nectin-4 expression. Immunofluorescence staining confirms Nectin-4 expression (green) in breast and bladder cancer cell lines. Nuclei are counterstained with DAPI (blue).

Histological and Immunohistochemical Staining of Tumor and Normal Tissues

Hematoxylin and eosin (H&E) staining of tumor xenografts derived from the aforementioned breast and bladder cancer cell lines confirmed viable tumor morphology with no signs of necrosis of hemorrhage (**Supplemental Figure 4A**). Immunohistochemical (IHC) staining for Nectin-4 revealed strong membrane-associated expression in MDA-MB-468, BT474, HT-1376, and SW780 tumors, consistent with their known Nectin-4-positive status (**Supplemental Figure 4B**). In contrast, MDA-MB-231 and UM-UC-3 tumors showed minimal or undetectable staining, confirming their Nectin-4-negative phenotype. To assess potential off-target expression and systemic toxicity, we further examined major organs by H&E and IHC staining. Histological examination showed preserved tissue architecture across various major organs with no evidence of inflammation or pathological damage following radiotracer administration (**Supplemental Figure 4C**). Corresponding IHC staining demonstrated some detectable staining in the lung, kidney, and intestine, suggesting low-level expression in these tissues (**Supplemental Figure 4D**). All other organs showed minimal to no Nectin-4 expression.

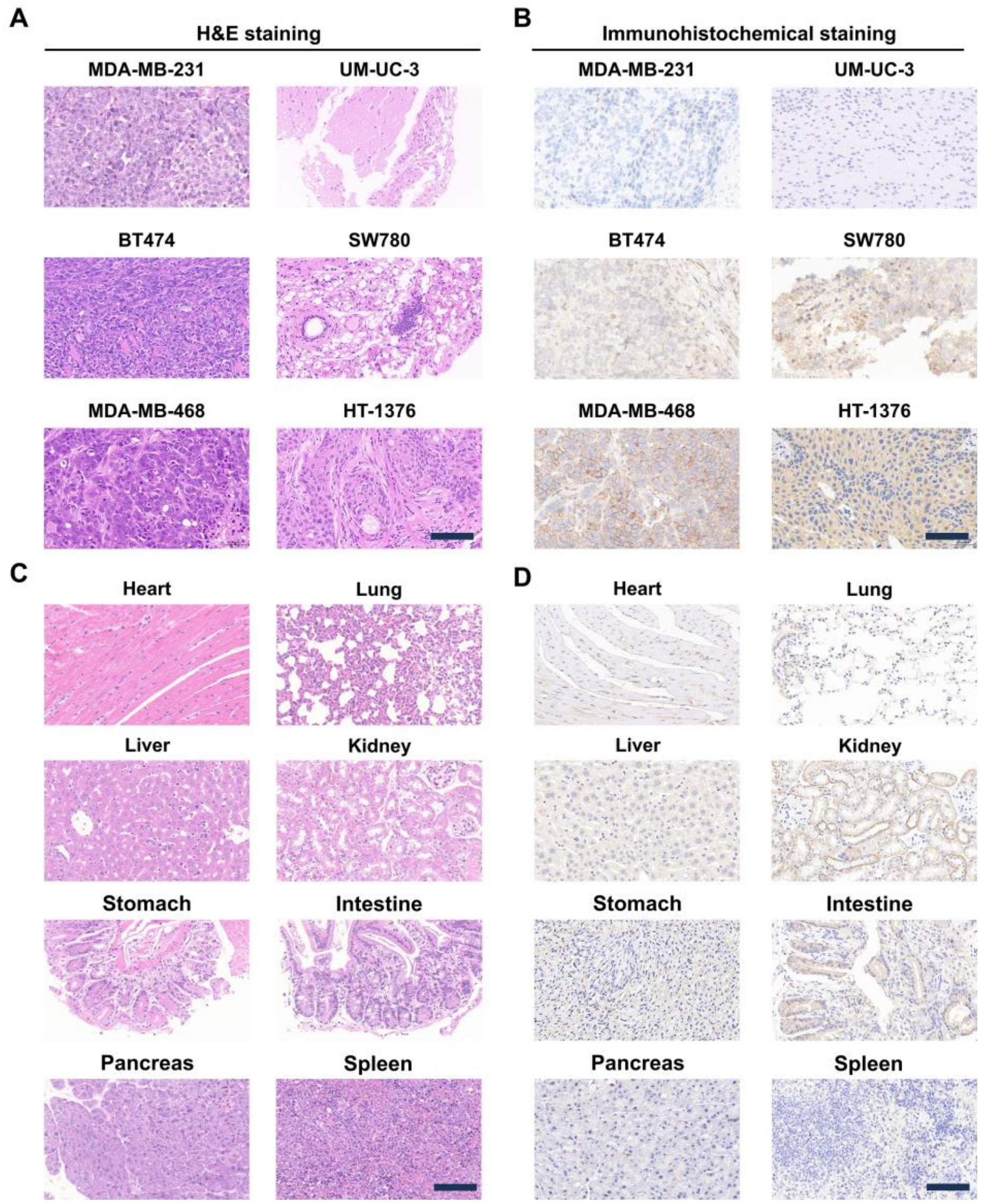

Supplemental Figure 4. Histological and immunohistochemical evaluation of Nectin-4 expression in tumor and normal tissues. (A) H&E staining of tumor sections from six cancer cell line–derived xenografts. **(B)** Immunohistochemical staining (IHC) for Nectin-4 expression in corresponding tumor tissues. **(C)** H&E and **(D)** IHC staining of various major organs.

Radiation dosimetry extrapolation to humans

Dosimetry analysis was conducted using OLINDA/EXM software. Estimated human dosimetry was derived from average %ID/g values obtained from serial PET scans on BALB/c mice, converted to %ID in humans. Assuming similar biodistribution in adult humans as in animal models, a monoexponential model was applied to the time–activity curves. OLINDA provides effective dose outputs, and weighting factors from the International Commission on Radiological Protection Publication 103 were utilized to convert these to absorbed doses for each organ. The estimated radiation doses of [^{64}Cu]Cu-NOTA-EV-F(ab')$_2$ for human organs, derived from biodistribution data, are summarized in **Supplementary Table 1**. Similarly, the radiation dose estimates for [^{64}Cu]Cu-NOTA-EV are provided in **Supplementary Table 2**. The estimated systemic effective dose for an adult woman was 0.0353 mSv/MBq, which falls within acceptable limits for conventional nuclear medicine research. The predictions should be regarded as preliminary estimates requiring further validation in clinical settings.

In preparation for potential clinical translation of [^{64}Cu]Cu-NOTA-EV-F(ab')$_2$, we carefully evaluated the specific activity, proposed dosing, and safety profile of the imaging agent. The specific activity achieved in this study was approximately 0.36 ± 0.02 MBq/μg, which is suitable for high-contrast imaging and would allow administration of microdoses in a clinical setting. Based on typical receptor expression levels and pharmacokinetic considerations, we propose a clinical mass dose of 100–300 μg per patient, corresponding to a total injected activity of 185–370 MBq (5–10 mCi), consistent with other clinically used ^{64}Cu-based immunoPET agents.

Regarding safety, the use of IdeS protease effectively removes the Fc region, which is the primary site of MMAE conjugation in Enfortumab vedotin. This process, combined with Fc-depletion purification steps, is expected to produce an imaging agent with negligible residual cytotoxic payload. While we did not directly quantify Monomethyl Auristatin E payload (MMAE) content in this study, the absence of adverse effects in

preclinical models supports the low toxicity profile of the final product.

For human use, GLP-compliant toxicology studies would be necessary to confirm safety. These studies should include mass spectrometry or HPLC analysis to verify the absence of MMAE, single-dose toxicity evaluations in large animal models, and detailed radiation dosimetry assessments using preclinical and human-derived biodistribution data. Given the low administered mass and anticipated lack of residual cytotoxicity, it is likely that this imaging agent could enter first-in-human studies under an exploratory IND (eIND) framework or a microdosing protocol, expediting its clinical evaluation.

These considerations highlight the strong translational potential of [^{64}Cu]Cu-NOTA-EV-F(ab')$_2$ as a safe and effective immunoPET agent for noninvasive assessment of Nectin-4 expression, supporting personalized treatment strategies for patients undergoing EV therapy.

Supplemental Table 1. Human organ radiation dosimetry estimation of [^{64}Cu]Cu-NOTA-EV-F(ab')$_2$.

Target Organ	mSv/MBq
Adrenals	3.77E-04
Brain	6.77E-05
Breasts	3.48E-03
Esophagus	1.28E-03
Eyes	0.00E00
Gallbladder Wall	3.42E-04
Left colon	1.79E-03
Small Intestine	4.08E-04
Stomach Wall	5.27E-03
Right colon	1.75E-03
Rectum	8.24E-04
Heart Wall	5.24E-04
Kidneys	1.86E-03
Liver	1.35E-03
Lungs	4.17E-03
Ovaries	1.44E-03
Pancreas	3.64E-04
Salivary Glands	3.00E-04
Red Marrow	6.11E-03
Osteogenic Cells	3.67E-04
Spleen	2.71E-04
Thymus	3.12E-04
Thyroid	1.24E-03
Urinary Bladder Wall	1.27E-03
Uterus	1.65E-04
Effective Dose	3.53E-02

Supplemental Table 2. Human organ radiation dosimetry estimation of [^{64}Cu]Cu-NOTA-EV

Target Organ	mSv/MBq
Adrenals	3.26E-04
Brain	7.40E-05
Breasts	3.43E-03
Esophagus	1.33E-03
Eyes	0.00E00
Gallbladder Wall	3.15E-04
Left colon	1.71E-03
Small Intestine	3.36E-04
Stomach Wall	4.31E-03
Right colon	1.65E-03
Rectum	7.79E-04
Heart Wall	9.28E-04
Kidneys	3.40E-04
Liver	1.98E-03
Lungs	5.52E-03
Ovaries	1.36E-03
Pancreas	2.07E-04
Salivary Glands	2.90E-04
Red Marrow	3.22E-03
Osteogenic Cells	2.55E-04
Spleen	3.47E-04
Thymus	3.24E-04
Thyroid	1.22E-03
Urinary Bladder Wall	1.21E-03
Uterus	1.57E-04
Effective Dose	3.16E-02
